# Handgrip strength and its prognostic value for mortality in Moscow, Denmark, and England

**DOI:** 10.1371/journal.pone.0182684

**Published:** 2017-09-01

**Authors:** Anna Oksuzyan, Panayotes Demakakos, Maria Shkolnikova, Mikael Thinggaard, James W. Vaupel, Kaare Christensen, Vladimir M. Shkolnikov

**Affiliations:** 1 Max Planck Institute for Demographic Research, Rostock, Germany; 2 Department of Epidemiology and Public Health, University College London, London, United Kingdom; 3 Scientific Institute of Pediatry at the Pirogov Moscow Medical University, Moscow, Russian Federation; 4 The Danish Twin Registry, Institute of Public Health, University of Southern Denmark, Odense, Denmark; 5 Max Planck Odense Center on the Biodemography of Aging, Institute of Public Health, Odense, Denmark; 6 Department of Clinical Genetics, Odense University Hospital, Odense, Denmark; 7 Department of Clinical Biochemistry and Pharmacology, Odense University Hospital, Odense, Denmark; 8 National Research University Higher School of Economics, Moscow, Russian Federation; University of Palermo, ITALY

## Abstract

**Background:**

This study compares handgrip strength and its association with mortality across studies conducted in Moscow, Denmark, and England.

**Materials:**

The data collected by the Study of Stress, Aging, and Health in Russia, the Study of Middle-Aged Danish Twins and the Longitudinal Study of Aging Danish Twins, and the English Longitudinal Study of Ageing was utilized.

**Results:**

Among the male participants, the age-standardized grip strength was 2 kg and 1 kg lower in Russia than in Denmark and in England, respectively. The age-standardized grip strength among the female participants was 1.9 kg and 1.6 kg lower in Russia than in Denmark and in England, respectively. In Moscow, a one-kilogram increase in grip strength was associated with a 4% (hazard ratio [HR] = 0.96, 95% confidence interval [CI]: 0.94, 0.99) reduction in mortality among men and a 10% (HR = 0.90, 95%CI: 0.86, 0.94) among women. Meanwhile, a one-kilogram increase in grip strength was associated with a 6% (HR = 0.94, 95%CI: 0.93, 0.95) and an 8% (HR = 0.92, 95%CI: 0.90, 0.94) decrease in mortality among Danish men and women, respectively, and with a 2% (HR = 0.98, 95%CI: 0.97, 0.99) and a 3% (HR = 0.97, 95%CI: 0.95, 0.98) reduction in mortality among the English men and women, respectively.

**Conclusion:**

The study suggests that, although absolute grip strength values appear to vary across the Muscovite, Danish, and English samples, the degree to which grip strength is predictive of mortality is comparable across national populations with diverse socioeconomic and health profiles and life expectancy levels.

## Introduction

Handgrip strength has been shown to predict all-cause and cause-specific mortality [1–5], old-age disability [6–8], cognitive decline [9], and hospitalization [10]. Because grip strength has these attributes, and because it is simple and inexpensive to measure, many surveys on health and mortality collect data on grip strength, and provide normative values of grip strength for various populations. However, as the growing literature on normative values suggests that they tend to differ across countries, caution is advised when applying these values internationally [11–15]. A recent systematic literature review of the published normative data for grip strength indicated that the average grip strength is substantially lower in developing regions than in developed countries [16].

Most of the previous international comparisons of grip strength and its prognostic abilities were carried out across countries with low mortality levels [17–19]. However, the results of recent research based on data from 17 countries appear to support the use of grip strength in stratifying the individual risk of death in countries with low to middle levels of economic development [20]. Yet to our knowledge, no previous studies have compared the grip strength levels and their ability to predict the stratification of the individual risk of survival of the Russian population with those of the populations of other countries.

It has been previously shown that the Russian mortality crisis is characterized by extremely high mortality levels from violence, accidents, and alcohol-related causes at working ages; and by high levels of cardiovascular disease (CVD) mortality at middle and older ages [21, 22]. From the mid-1960s to the early 2000s Russia experienced adverse mortality trends particularly in times of the socioeconomic crisis in the early 1990s with a few short-lived interruptions of these downward trends in mid-1980s due to a wide-ranging anti-alcohol campaign by Mikhail Gorbachev and 1995–97[23, 24]. In 2004–2015, the country experienced improvements in life expectancy, largely due to substantial reductions in mortality from external causes at working ages and circulatory diseases at older ages[22, 25, 26]. Despite these recent gains, there is still a huge 10-year life expectancy gap between Russia and the western countries.

In addition to suffering from excess mortality, Russians have relatively low levels of self-rated health and of physical functioning, especially at older ages [27–30]. However, the existing studies did not show that objectively measured conventional CVD risk factors, e.g. blood pressure and blood lipids, are substantially worse in Russia than they are in low-mortality countries [31–35]. Although the prevalence levels of metabolic syndrome are relatively high among women in Russia, they are still lower than the prevalence levels found among their counterparts in the USA, and they are comparable to the prevalence levels observed in other countries with much lower mortality [36, 37].

Nearly nothing is known about how well Russians perform on grip strength tests relative to other Europeans, or have examined whether grip strength can be used to stratify individual survival risk in the Russian population, as it has in western European (EU) populations. Thus, in the present study we aim to compare the levels of handgrip strength across studies conducted in Moscow, Denmark, and England. We also compare the value of grip strength for predicting mortality in the Russian, Danish, and English populations.

The steady decline in both male and female mortality from CVD and other major causes in the UK resulted in a widening of the life expectancy gap between Russia and the UK over the period from 1970 to 2013, from 5.6 years to 13.9 years among men, and from 1.6 years to 6.5 years among women [22, 26, 38]. In addition, the UK has been shown to have life expectancy levels and causes of death that are close to the EU averages [26], and normative grip strength values that are slightly above the mean levels across 11 EU countries [13, 39]. Relative to other EU countries, Denmark has a below-average life expectancy level [40], but still much higher than in Russia (7.0 years among women and 13.2 years among men in 2013). Denmark has also one of the highest grip strength scores [39].

While it is difficult to predict how similar the prognostic importance of grip strength will prove to be across these three socio-culturally and economically distinct settings, in our study samples we expect to find that the Russians scored worse on grip strength tests than the English, and far worse than the Danes. Considering a strong sex-specific disconnect between health and survival in the Russian population [27, 41], we expect to find a greater male advantage in grip strength in the Russian than in the Danish and the English study samples. In light of previous research showing that grip strength predicts mortality at middle and older ages to similar degrees in both sexes [42, 43], we hypothesize that the predictive power of grip strength will be similar among the men and the women studied in Moscow, Denmark, and England.

## Materials and methods

### Study populations

The study utilized data collected by the Stress Aging and Health in Russia (SAHR) study, by the Study of Middle-Aged Danish Twins (MADT) and the Longitudinal Study of Aging Danish Twins (LSADT), and by the English Longitudinal Study of Ageing (ELSA). Detailed descriptions of these studies have been provided elsewhere [44–47].

The SAHR is a prospective population-based cohort study of Muscovites aged 55 and older. The participants were randomly selected from seven epidemiological cohorts who were identified in the Lipid Research Clinics (LRC) and the MONICA studies conducted from the mid-1970s through the 1990s [46, 48]. These cohorts were randomly sampled from two typical districts of Moscow and served as the central source of information about cardiovascular risk factors and their links to mortality in the Russian population [30–32, 35]. Since these epidemiological cohorts consisted primarily of people who were living in Moscow before the mid-1980s, additional participants who had moved to Moscow after 1985 were identified from the Moscow Outpatient Clinics registry and added to the sample. The SAHR baseline survey, which was conducted between December 2006 and June 2009 and had a 66% response rate, included 1,800 participants. Although it is a population-based sample of the Moscow population, the SAHR sample differs in some respects from the entire Russian population. The most important difference is that Moscow has a greater proportion of people with higher education and higher income. The weights by age and education were estimated and are used to bring the SAHR sample closer to the national population for cross-country comparison of levels of grip strength.

Face-to-face interviews and extensive medical examinations were performed, mainly at the hospital; although a small share (8%) of the participants who were unable or reluctant to come to the hospital were interviewed at home using the hospital protocol. A substantial portion of the SAHR questionnaire was modeled on the data collection instrument used in the LSADT. The analytical sample consisted of 1,781 (54% women), after five individuals were excluded because they were not between the ages of 55 and 89, and 14 individuals were excluded because of missing data on grip strength.

Eligible participants from the MADT and the LSADT were identified through the Danish Twin Register, which is the oldest twin register worldwide [49, 50]. The MADT represented a random sample of 120 twin pairs from each birth year between 1931 and 1952, and who were thus aged 45–68 in 1998 when the baseline assessment was carried out. Of these 5280 individual twins in the sampling framework, 90 died before the time the survey was undertaken, and 4314 (83%) of the 5190 surviving twins participated in a personal interview and a health examination.

The LSADT covers twins born between 1909 and 1930. The intake survey included twins who were aged 75 or older and were living in Denmark in January 1995. Follow-up waves were conducted every second year until 2005, and new participants aged 70 or older were added in 1997, 1999, and 2001. Because the LSADT data on grip strength were first collected in the second follow-up survey in 1999, for our analysis we considered only the LSADT survey data from 1999 for existing participants and the survey data from 2001 for newly added participants (n = 2,769). After excluding 1,942 individuals who were not in the 55–89 age range at the intake examination and 354 individuals for whom grip strength measurements are missing from the combined MADT and LSADT participants (n = 7,483), our study sample consisted of 5,187 twins (2,688, or 52% of whom were women). Although the age distributions of their study populations differed slightly, the MADT and the LSADT are comparable in terms of their design, implementation, and data collection instruments. The data collection in each survey wave was carried out at the participants’ homes, and no exclusion criteria were used to select eligible respondents. If an individual refused to or was unable to participate in the face-to-face interview, a proxy respondent, usually a close relative, was sought. The response rates at the baseline surveys were 83% in the MADT and 77% in the LSADT [44, 45].

The respondents of the baseline LSADT were similar to the non-respondents in terms of age distribution, zygosity, and early hospitalizations in 1977–94 [44]; although women who had been hospitalized within the last two years and women who had been using prescription medicine within the six months before and after the intake surveys were slightly overrepresented in the surveys [51]. Previous research in Denmark, Sweden, and the USA has indicated that twins are representative of the general population with respect to all-cause and cardiovascular mortality; morbidity due to diabetes, cancer, and CVD; and lifestyle characteristics [52–56].

The English Longitudinal Study of Ageing (ELSA) is a prospective study of community-dwelling residents of England aged 50 or older. At its launch in 2002–03, the ELSA had a nationally representative sample of 11,391 participants. The participants were recruited from nationally representative samples that had been used earlier by the Health Survey for England, and were selected through a multi-staged stratified random probability design. A detailed profile of the ELSA can be found at: http://www.elsa-project.ac.uk/. The ELSA collects interview and health examination data at regular two- and four-year intervals, respectively. The first wave of health examination data was collected during the first follow-up in 2004–05. The analytical sample consisted of 5,852 individuals (3,084, or 53% of whom were women) who were selected among the 7,666 individuals who consented to take part in a health examination in 2004–05 (out of an initial sample of 8,780 individuals who took part in the first wave of follow-up interviews in 2004–05), and after 704 participants were excluded because they were outside of the 55–89 age range, and 1,114 individuals were excluded because of missing or incomplete data. Among all of the individuals eligible to participate, the response rate in the first follow-up examination in 2004–05 was 82%. The response rate for the health examination was 71% among all of the individuals who were eligible to participate in the first follow-up in 2004–05, and was 88% among all of the individuals who were interviewed in the first follow-up in 2004–05 [47, 57].

### Grip strength

In all three settings, grip strength in kilograms was measured by using a Smedley dynamometer (TTM; Tokyo, Japan), with the upper arm being held against the trunk and the elbow in a 90-degree flexion [15, 46](see http://bit.ly/1TeAJwU). Three trials with brief pauses were performed for each hand, and the subjects are encouraged to exert the maximal effort. The participants who made fewer than three attempts, or for whom there was a difference of 20 kg or more between the two measures, were excluded. The maximal handgrip strength of six measures was used in the present analyses.

The percentage of missing observations on grip strength due to the exclusion criteria and proxy respondents was 5.8%. In the Danish surveys, missing values on grip strength were related to being a woman, being older, reporting poor general health, and having lower cognitive functioning as measured by an immediate recall test. Information on grip strength was missing for 14 individuals (0.8%) in the SAHR, 354 twins in the Danish samples (4.7% of all of the 7,483 intake participants), and 113 individuals in the ELSA (1.5% of the 7,666 individuals who participated in the health examination in 2004–05).

### Potential confounders

In the Danish surveys, height and weight were collected through self-reports. In the SAHR, a wall-mounted stadiometer and calibrated scales were used to measure body height and weight, respectively. In the ELSA, nurses measured the participants’ weight without shoes and any bulky clothing using a portable stadiometer, and their standing maximum height in the Frankfort plane using a portable electronic scale.

In all three studies, the participants’ educational level was categorized as primary, secondary/middle, or high. In the Danish surveys the participants’ educational level was ascertained by asking them two questions: one about the type of elementary school they attended, and another about the level of vocational education they received after leaving elementary school. In the SAHR similar information about the participants’ educational level was collected using one question. The ELSA collected information on educational attainment, which was used to derive the education variable. Individuals with foreign educational qualifications were excluded from the present analyses.

Smoking status was defined as never smoker, former smoker, or current smoker. Cognitive function was assessed using a recall test that asked participants to recall immediately a list of 12 nouns (in the SAHR and the LSADT) or 10 words (in the ELSA) [58, 59]. The total number of correctly recalled words was computed as a total score. To assess their history of chronic diseases and conditions, the participants were asked whether they had been ever told by a doctor that they had any of a list of diseases, with the response options being “no,” “have had,” and “have now” in the SAHR and the LSADT 1999 and 2001; and “yes” and “no” in the LSADT 1995 and 1997. The number of chronic conditions was calculated on the basis of this list of 14 conditions (diabetes, chronic bronchitis, Parkinson’s disease, cancer, stroke, myocardial infarction, angina, arrhythmia, hypertension, heart failure, hyperthyroidism, rheumatoid arthritis, osteoporosis, and asthma). In the ELSA, no information on thyroid disease was collected.

### Mortality follow-up

To ensure that the maximum length of the follow-up in the three study populations was similar, vital status was ascertained through January 2014 in Moscow; through January 1, 2006, in Denmark via the linkage to the Central Personal Register; and through February 2013 in the ELSA. The average follow-up time was 6.2 years (range: 0.01, 7.21) in Moscow, 5.9 years (range: 0.01, 7.19) in the MADT and the LSADT, and 7.5 years in the ELSA.

Due to the additional exclusion of individuals with missing values on confounders, the analytical sample for the survival analysis consisted of 1,739 participants (956 women, 55%) in Moscow, 5,041 (2,619 women, 52%) participants in Denmark, and 5,852 (3,084 women, 53%) participants in England. By the end of the follow-up period, there were 288 deaths (17% of the study sample, 192 (67%) men) in the SAHR, 974 (19% of the study sample, 532 (55%) men) deaths in the MADT and the LSADT, and 925 (16%, 518 (56%) men) deaths in the ELSA.

### Statistical analysis

Since the age distribution differed across the studies, we restricted the age range in our analysis to 55–89. We performed direct age standardization using the standard European population in order to compare overall grip strength across the countries [60, 61]. In the cross-country comparison of grip strength, we used post-stratification weights that adjust for differences in age and education (within each sex) between the sample and the Russian population (based on the 2002 census). The survival analysis was based on unweighted data.

Cox proportional hazard regression models were used to estimate the change in mortality for every kilogram change in grip strength in the total and sex-specific samples. In all of the models we used age as the time scale, and assessed the risk from the age at the measurement of grip strength to the age at death or the end of the follow-up, whichever came first. Adjustments were made for height, weight, education, and smoking status. A sensitivity analysis was performed by adding to the model immediate recall, a number of chronic conditions, or self-rated health.

A two-tailed p-value of less than 0.05 was considered to be significant. All of the analyses were performed using STATA, version 14.0 [62]. The proportional hazards assumption for each covariate was tested on the basis of Schoenfeld residuals after fitting a model. To account for within-twin-pair similarity, robust standard errors were estimated that allow clustering within twin pair and that assume independence of each pair [63].

The study involves secondary data analysis of existing survey data. The LSADT and the MADT were approved by the ethical committee assigned through the Danish National Committee on Biomedical Research and the Danish Data Protection Agency. The SAHR was approved by the Ethical Committee of the State Research Centre for Preventive Medicine, Moscow, Russia; and by the Institutional Review Board at Duke University, Durham, USA. Ethical approval for all of the ELSA waves was granted by the National Research Ethics Service.

## Results

Table 1 shows characteristics of the SAHR, the MADT, the LSADT, and the ELSA samples. On average Muscovite women were heavier than Danish and English women, and Muscovite men and women were older and more educated than their Danish and English counterparts. It should be noted, however, that the comparison of education levels across different settings should be done with caution as similar educational levels may have different definitions and may imply different levels of wealth and prestige within each country. The percentages of women who were never smokers and the percentage of participants who had at least one chronic disease were much higher in Moscow than in Denmark and England.

**Table 1:** Descriptive characteristics of the Muscovite, Danish, and English samples.

	SAHR				MADT and LSADT				ELSA			
	Men		Women		Men		Women		Men		Women	
	N*	Mean (SE)	N	Mean (SE)	N	Mean (SE)	N	Mean (SE)	N	Mean (SE)	N	Mean (SE)
Age	835	69.3 (0.28)	960	68.2 (0.23)	3585	64.5 (0.19)	3898	66.4 (0.20)	2768	66.9 (0.16)	3084	67.4 (0.16)
Height (cm)	834	172 (0.23)	958	159 (0.20)	2620	174 (0.13)	2910	163 (0.11)	2768	173 (0.13)	3084	159 (0.12)
Weight (kg)	834	81 (0.51)	958	74 (0.43)	2616	78 (0.22)	2894	64 (0.22)	2768	83 (0.27)	3084	71 (0.26)
	**N**	**%**	**N**	**%**	**N**	**%**	**N**	**%**	**N**	**%**	**N**	**%**
**Education**												
Primary	94	11.6	73	7.7	789	31.1	1488	52.0	951	34.4	1512	49.0
Secondary	326	40.3	384	40.6	1246	49.0	857	30.0	930	33.6	890	28.9
High	389	48.1	489	51.7	506	19.9	516	18.0	887	32.0	682	22.1
**Smoking status **												
Never smoked	279	33.5	773	80.5	518	19.8	1250	43.0	951	34.4	1512	49.0
Ex-smoker	342	41.1	106	11.0	1079	41.2	765	26.3	930	33.6	890	28.9
Current smoker	212	25.5	81	8.4	1021	39.0	893	30.7	887	32.0	682	22.1
**No. of chronic conditions **												
None	98	11.8	56	5.8	1274	48.6	1200	41.2	709	25.6	681	22.1
1	159	19.1	120	12.5	671	25.6	876	30.0	871	31.5	940	30.5
2	162	19.5	193	20.1	379	14.5	482	16.5	650	23.5	756	24.5
3+	412	49.6	590	61.5	299	11.4	358	12.3	538	19.4	707	22.9

* N–sample size, SE–standard error, SAHR–Study of Stress, Aging, and Health in Russia, MADT–the Study of Middle-Aged Danish Twins, LSADT–the Longitudinal Study of Aging Danish Twins, ELSA–the English Longitudinal Study of Ageing

**Fig 1:**
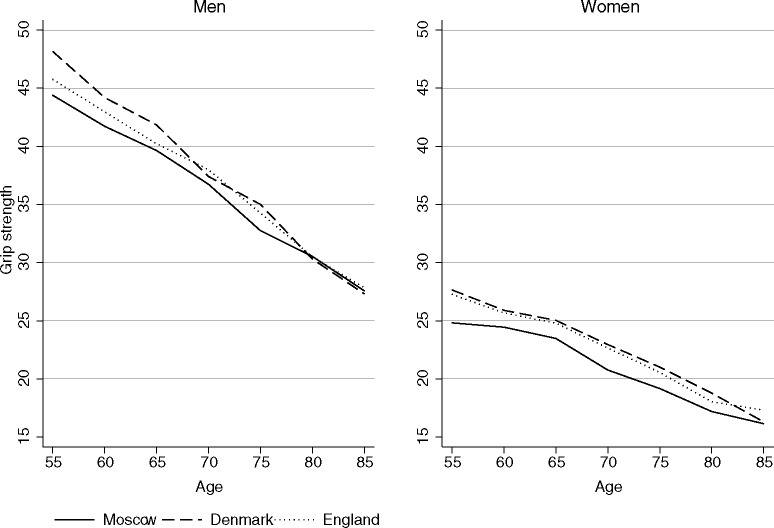
Cross-sectional age trajectories of grip strength in Moscow, Denmark, and England.

The men in Moscow had lower levels of grip strength than the men in Denmark except at the ages 80–89 years. At all ages, except 80 year and above, Muscovite men performed somewhat worse than their English counterparts. The corresponding gap was statistically significant for the ages 70–74 years and for all ages combined (Table 2 and Fig 1). The women in Moscow had substantially less grip strength than the women in Denmark and England at all ages, except in the age groups 80–84 and 85–89. The age-standardized grip strength among the male SAHR participants was about 2 kg and 1 kg lower than that of the Danish male twin participants and the ELSA male participants, respectively. The age-standardized grip strength of the women in Moscow was 1.9 kg lower than that of the women in Denmark, and was 1.6 kg lower than that of the women in England. The cross-country gaps became 0.2 kg greater when the high educational levels of the Muscovite sample were accounted for. The gender difference in grip strength favoring men was substantial, and was similar across the three settings: 16.3 kg in the SAHR, 16.4 kg in the MADT and the LSADT, and 15.7 kg in the ELSA.

**Table 2:** Grip strength in Moscow, Denmark, and England.

	SAHR^a^			MADT and LSADT			ELSA			SAHR education-adjusted		
**Men**												
**Age**	**N**	**Mean**	**SE**	**N**	**Mean**	**SE**	**N**	**Mean**	**SE**	**N**	**Mean**	**SE**
55–59	125	44.4	0.68	501	48.2*^c^	0.32	689	45.8	0.31	125	44.2	0.67
60–64	159	41.7	0.57	470	44.2*^c^^c^	0.35	527	43.0	0.39	159	41.5	0.58
65–69	158	39.6	0.56	294	41.8**^c^	0.43	538	40.2	0.35	158	39.6	0.56
70–74	160	36.7	0.52	679	37.4	0.29	448	38.0^ǂ^^d^	0.34	160	36.7	0.52
75–79	127	32.7	0.66	338	35.0**^c^	0.40	311	34.3	0.40	127	32.5	0.66
80–84	68	30.5	0.71	131	30.3	0.62	186	30.5	0.53	68	30.4	0.71
85–89	28	27.6	1.46	86	27.3	0.75	71	27.8	0.90	28	27.0	1.47
All ages	825	38.0	0.29	2499	40.3	0.19	2770	40.1	0.18	825	37.8	0.30
**Age-stand.**^**b**^	825	38.4	0.25	2499	40.4*^c^	0.15	2770	39.4*^d^	0.15	825	38.2	0.25
**Women**												
**Age**	**N**	**Mean**	**SE**	**N**	**Mean**	**SE**	**N**	**Mean**	**SE**	**N**	**Mean**	**SE**
55–59	146	24.8	0.44	462	27.7*^c^	0.26	756	27.3*^d^	0.22	146	24.9	0.42
60–64	200	24.5	0.33	472	25.9*^c^	0.24	593	25.7**^d^	0.24	200	24.4	0.34
65–69	227	23.5	0.30	270	25.0*^c^	0.31	556	24.8*^d^	0.23	227	23.3	0.31
70–74	217	20.8	0.32	677	23.0*^c^	0.20	439	22.7*^d^	0.25	217	20.4	0.33
75–79	87	19.2	0.53	410	21.0**^c^	0.24	370	20.6^ǂ^^d^	0.27	87	18.6	0.50
80–84	66	17.2	0.62	249	18.8	0.28	259	18.0	0.31	66	16.8	0.59
85–89	13	16.2	1.15	148	16.3	0.37	105	17.3	0.41	13	15.0	1.27
All ages	956	22.3	0.17	2688	23.4	0.12	3078	23.9	0.11	956	21.9	0.18
**Age-stand.**	956	22.1	0.16	2688	24.0*^c^	0.10	3078	23.7*^d^	0.10	956	21.9	0.15

a: N–sample size, SE–standard error, SAHR–Study of Stress, Aging, and Health in Russia, MADT–the Study of Middle-Aged Danish Twins, LSADT–the Longitudinal Study of Aging Danish Twins, ELSA–the English Longitudinal Study of Ageing

b: Standardized to the Revised European Standard Population 2013: http://www.ons.gov.uk/ons/guide-method/user-guidance/health-and-life-events/revised-european-standard-population-2013--2013-esp-/index.html

c: p–value for SAHR vs. DK differences in grip strength

d: p–value for SAHR vs. ELSA differences in grip strength

* p-value < 0.001

** p-value <0.01

^ǂ^ p-value <0.05

Table 3 presents the hazard ratios (HRs) for grip strength in total and sex-specific samples for Moscow, Denmark, and England. In the base model adjusted for gender, height and weight, a one-kilogram increase in grip strength was associated with a 5% (hazard ratio [HR] = 0.95, 95% confidence interval [CI]: 0.93, 0.96), a 6% (HR = 0.94, 95%CI: 0.93, 0.95), and a 3% (HR = 0.97, 95% CI: 0.96, 0.98) mortality reduction in the Moscow, Danish, and English total samples, respectively (Table 3). A one-kilogram increase in grip strength was associated with a 4% (HR = 0.96, 95%CI: 0.94, 0.98) and a 10% (HR = 0.90, 95% CI: 0.86, 0.94) reduction in mortality among the Russian men and women, respectively (Table 3). Similarly, HR of mortality per one-kilogram increase in grip strength was 0.94 (95% CI: 0.93, 0.95) among the Danish men and 0.93 (95% CI: 0.91, 0.95) among the Danish women.

**Table 3:** Mortality per 1-kg increase in grip strength in Moscow, Denmark, and England.

	SAHR^a^				MADT and LSADT				ELSA			
	HR	95%CI		p-value	HR	95%CI		p-value	HR	95%CI		p-value
**Total sample**												
**Model 1**^**b**^												
Grip	0.95	0.93	0.96	<0.001	0.94	0.93	0.95	<0.001	0.97	0.96	0.98	<0.001
**Model 2**												
Grip	0.95	0.94	0.97	<0.001	0.94	0.93	0.95	<0.001	0.98	0.97	0.99	<0.001
**Men**												
**Model 1**												
Grip	0.96	0.94	0.98	<0.001	0.94	0.93	0.95	<0.001	0.97	0.96	0.99	<0.001
**Model 2**												
Grip	0.96	0.94	0.99	0.001	0.94	0.93	0.95	<0.001	0.98	0.97	0.99	<0.001
**Women**												
**Model 1**												
Grip	0.90	0.86	0.94	<0.001	0.93	0.91	0.95	<0.001	0.96	0.94	0.98	<0.001
**Model 2**												
Grip	0.90	0.86	0.94	<0.001	0.92	0.90	0.94	<0.001	0.97	0.95	0.98	<0.001

^**a**^ HR–hazard ratio, CI–confidence interval, SAHR–Study of Stress, Aging, and Health in Russia, MADT–the Study of Middle-Aged Danish Twins, LSADT–the Longitudinal Study of Aging Danish Twins, ELSA–the English Longitudinal Study of Ageing

^**b**^ Model 1: Grip + height, weight, and gender (in the total sample only); Model 2: Grip + height, weight, education, smoking status, and gender (in the total sample only)

**Fig 2:**
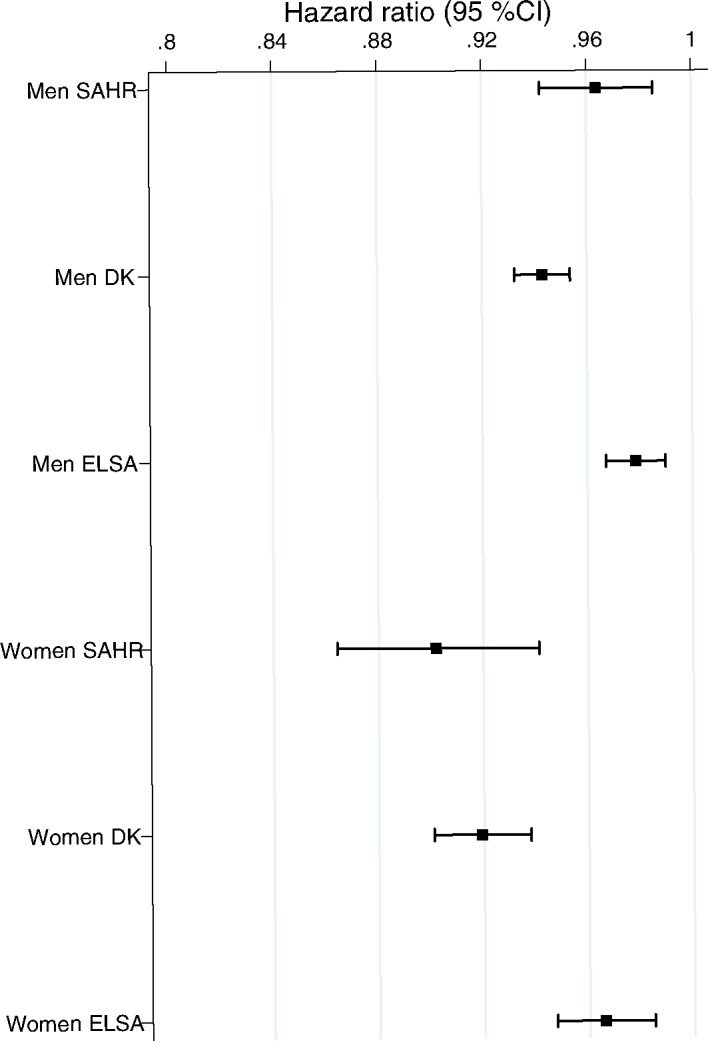
Hazard ratios for grip strength per 1-kg increase in SAHR, MADT and LSADT, and ELSA.

The predictive ability of grip strength was slightly lower in the ELSA: it was 3% (HR = 0.96, 95% CI: 0.96, 0.99) among the men and 4% (HR = 0.96, 95%CI: 0.94, 0.98) among the women. The predictive value of grip strength remained essentially unchanged in all three settings after successive adjustments were made for education and smoking (Model 2, Table 3 and Fig 2), as well as when cognitive functioning, the number of chronic conditions, and self-rated health were included into the models (S1 and S2 Tables). The associations between grip strength and mortality were similar among the men and the women in Denmark and in England, while in Moscow the reduction in mortality for grip strength per one-kilogram increase was larger among the women than among the men.

## Discussion

The results of the present study suggest that the grip strength levels of the study participants in Moscow were lower than those of the study participants in Denmark and in England, and this disadvantage was more pronounced at younger than at older ages. These findings point toward possible influence of mortality selection, suggesting that in a population exposed to much higher mortality, such as Moscow, survivors to old ages are becoming relatively stronger compared to lower mortality populations. It has previously been shown that Danish men and women have very high scores on grip strength test at middle and older ages [17, 64]. Genetic factors and gene-environment interactions have been suggested as possible explanations for cross-country differences in muscle strength. The study participants in Moscow had lower grip strength than their counterparts in most of the countries included in the Survey of Health, Ageing and Retirement in Europe (SHARE)[39]. According to the SHARE data, only the participants in Spain and in Italy had similar or slightly lower levels of grip strength than the participants in Moscow. Our finding that the SAHR participants had less grip strength than the LSADT, the MADT, and the ELSA participants is in line with the results of previous studies that showed that people living in Russia were in worse reported health than people living in Central and Eastern European countries or in Sweden [28–30].

A recent study in the North-West Russia has attempted to provide normative values of grip strength in the Russian population [65]. The levels of grip strength indicated among SAHR participants are lower than those reported by this study [65]. It should be noted, however, the study has a very small sample of 611 people aged 65 years and older selected from one polyclinic and utilized a non-standard dynamometer. The authors provide no information about the recruitment procedure and response rate, as well as they do not report or adjust for educational composition of the sample.

Interestingly, we found that the Danish men and women had more grip strength than their English counterparts, even though life expectancy was higher in the UK than in Denmark (by 0.97 years for men and by 0.76 years for women) in 2005, when wave 2 of the ELSA was initiated [38]. This outcome is in line with the findings of a recent study conducted in 13 European countries and in the United States showing that the countries with the highest life expectancy at age 80 are not necessarily the countries with better health [66]. The results from the SHARE study demonstrate that there is a clear increase in grip strength levels from the south to the north of Europe that does not correspond with the current geographical mortality patterns at older ages [39]. Taken together, these findings suggest that the correlation across countries between grip strength and life expectancy is rather weak.

Our analyses showed that the mortality reduction per one-kilogram increase in grip strength in the male samples was slightly higher in Denmark than in the other countries, and was lowest in England. In the female samples, the mortality reduction per one-kilogram increase in grip strength was slightly higher in Moscow than in the other countries, and was again lowest England. However, the overlapping 95% CIs suggest that even though there were substantial differences between Russia and Denmark in terms of their grip strength levels, their economic development levels, their health care and social systems, and their cultures; the associations between grip strength and mortality were similar in Moscow and Denmark. The associations between grip strength and mortality in England differed slightly from those in Denmark (both men and women) and among the SAHR participants (men only). In the present study, the HRs estimated for the male populations were very close to the HR of 0.97 (95% CI: 0.96, 0.98) per one-kilogram increase of grip strength found in a meta-analysis study [19]. Our results for the ELSA female participants were similar to the estimates provided by Cooper et al., although we found that the mortality reduction was slightly higher among the women in Moscow. These discrepancies may be due to methodological differences, as some of the studies included in the meta-analyses did not provide sex-specific estimates [19].

The study of grip strength in the North-West Russia showed that in the model adjusted for age, sex, and comorbidity the hazard ratio for the lowest decile of maximum grip strength was 1.60 (95% CI: 1.14, 2.24), which is comparable to the estimates found in the SAHR sample (HR = 2.39 95% CI: 1.14, 5.02 analysis not shown here) despite methodological differences between these two studies concerning sample selection, measurement tool, and adjustment for covariates [65].

Our expectation that the gender difference in grip strength would be greater in Moscow than in Denmark or in England was premised on previous findings indicating that women in Moscow are more disadvantaged in terms of their general health, physical functioning, and depression symptomatology than women in Denmark [41]. Surprisingly, we found that the male-female difference in grip strength was similar across the study populations in Moscow, Denmark, and England. This outcome may suggest that the magnitudes of the gender differences in objective health measures are more similar across populations than they are in subjective measures. Furthermore, our finding that the associations between grip strength and mortality are similar among men and women is consistent the results of previous empirical studies on middle-aged and older populations[42, 43]. However, our finding of 95% CIs of hazard ratios for grip strength for the women in Moscow is rather wide due to the small number of deaths, and the male-female difference in the prognostic value of grip strength for mortality may have become more apparent if the follow-up had been longer.

Whether the available country-specific data are comparable is one of the key concerns researchers have when conducting comparison studies. Although the same devices and protocols were used to measure grip strength in the three studies, some portion of the variation in grip strength may be due to differences in the response rates and in the data collection procedures. For example, in Moscow and in Denmark the interviews and the performance tests were conducted in a single day, whereas in England the main interview was followed by a health examination, which was conducted by a nurse visit. The approach used in England may have resulted in a higher degree of selectivity in the study, which could have to some extent influenced the levels and predictive ability of grip strength. Although reported chronic conditions were much more prevalent among the participants in Moscow than among the participants in Denmark or in England, the adjustments for cognitive function and number of chronic conditions barely modified the predictive ability of grip strength in the three study settings (S1 Table). It is possible that some residual confounding may have affected this association, despite our attempts to adjust for several potential confounders. Furthermore, due to insufficient power it was not possible to investigate the question of whether the association between grip strength and mortality changes with age. However, a recent study conducted in Norway showed that the associations were similar across age groups and in both genders[42]. Our findings are based on a large-population-based sample of the general Moscow population. Although weighting allows adjusting for the differences in the educational structure between the study sample and the total Russian population, it is still questionable how generalizable are our results to Russia as a whole.

The present study adds to a growing body of research evidence indicating that grip strength is a powerful predictor of mortality across individuals, and that its predictive abilities are comparable across countries with diverse socioeconomic conditions, cultural circumstances, health profiles, and life expectancy levels. Despite the apparent differences in levels of grip strength, we found that the grip strength-mortality associations in Moscow were quite similar to those in Denmark and in England.

## Supporting information

S1 Table Hazard ratios for grip strength per 1-kg increase in total and gender-specific samples of Muscovite, Danish, English populations. (DOCX)

S2 Table Hazard ratios for grip strength per 1-kg increase in men and women in Moscow, Denmark, and England. (DOCX)
