# Supplementary material for: Handgrip strength and its prognostic value for mortality in Moscow, Denmark, and England
Source: PLoS One. 2017 Sep 1;12(9):e0182684. doi: 10.1371/journal.pone.0182684 (PMC5580990; doi:10.1371/journal.pone.0182684)
Supplement: S1 Table — (DOCX) [file pone.0182684.s001.docx]

**S1 Table. Hazard ratios for grip strength per 1-kg increase in total and gender-specific samples of Muscovite, Danish, English populations**

|  | **SAHR^a^** | | | | **MADT and LSADT** | | | | **ELSA** | | | |
| --- | --- | --- | --- | --- | --- | --- | --- | --- | --- | --- | --- | --- |
| **Total sample** | **HR** | **95%CI** |  | **p-value** | **HR** | **95%CI** |  | **p-value** | **HR** | **95%CI** |  | **p-value** |
| **Model 1** |  |  |  |  |  |  |  |  |  |  |  |  |
| Grip | 0.95 | 0.93 | 0.96 | <0.001 | 0.94 | 0.93 | 0.95 | <0.001 | 0.97 | 0.96 | 0.98 | <0.001 |
| Men | 4.14 | 2.78 | 6.15 | <0.001 | 4.01 | 3.31 | 4.86 | <0.001 | 2.45 | 1.99 | 3.02 | <0.001 |
| Height | 1.02 | 1.00 | 1.04 | 0.109 | 1.01 | 1.00 | 1.02 | 0.027 | 1.00 | 0.99 | 1.01 | 0.829 |
| Weight | 1.00 | 0.99 | 1.01 | 0.510 | 0.99 | 0.98 | 0.99 | 0.001 | 1.00 | 0.99 | 1.00 | 0.261 |
| **Model 2** |  |  |  |  |  |  |  |  |  |  |  |  |
| Grip | 0.95 | 0.94 | 0.97 | <0.001 | 0.94 | 0.93 | 0.95 | <0.001 | 0.98 | 0.97 | 0.99 | <0.001 |
| Men | 3.20 | 2.10 | 4.86 | <0.001 | 3.76 | 3.10 | 4.55 | <0.001 | 2.21 | 1.79 | 2.73 | <0.001 |
| Height | 1.02 | 1.00 | 1.04 | 0.114 | 1.01 | 1.00 | 1.02 | 0.039 | 1.00 | 0.99 | 1.01 | 0.716 |
| Weight | 1.00 | 0.99 | 1.01 | 0.865 | 0.99 | 0.98 | 1.00 | 0.013 | 1.00 | 0.99 | 1.00 | 0.504 |
| Education (ref.: low) | | |  |  |  |  |  |  |  |  |  |  |
| Middle | 0.80 | 0.57 | 1.12 | 0.198 | 0.89 | 0.77 | 1.03 | 0.107 | 0.78 | 0.66 | 0.92 | 0.003 |
| Higher | 0.70 | 0.49 | 1.00 | 0.048 | 0.79 | 0.66 | 0.96 | 0.016 | 0.79 | 0.66 | 0.94 | 0.008 |
| Smoking (ref.: never and ex-smoker) | | | | |  |  |  |  |  |  |  |  |
| Current | 2.01 | 1.48 | 2.72 | <0.001 | 1.59 | 1.39 | 1.83 | <0.001 | 2.07 | 1.74 | 2.46 | <0.001 |
| **Men** | **HR** | **95%CI** |  | **p-value** | **HR** | **95%CI** |  | **p-value** | **HR** | **95%CI** |  | **p-value** |
| **Model 1** |  |  |  |  |  |  |  |  |  |  |  |  |
| Grip | 0.96 | 0.94 | 0.98 | <0.001 | 0.94 | 0.93 | 0.95 | <0.001 | 0.97 | 0.96 | 0.99 | <0.001 |
| Height | 1.02 | 0.99 | 1.05 | 0.120 | 1.02 | 1.00 | 1.03 | 0.012 | 1.01 | 0.99 | 1.03 | 0.188 |
| Weight | 0.99 | 0.98 | 1.00 | 0.205 | 0.99 | 0.98 | 1.00 | 0.031 | 0.99 | 0.99 | 1.00 | 0.131 |
| **Model 2** |  |  |  |  |  |  |  |  |  |  |  |  |
| Grip | 0.96 | 0.94 | 0.99 | 0.001 | 0.94 | 0.93 | 0.95 | <0.001 | 0.98 | 0.97 | 0.99 | <0.001 |
| Height | 1.02 | 0.99 | 1.05 | 0.130 | 1.02 | 1.00 | 1.03 | 0.025 | 1.01 | 1.00 | 1.03 | 0.119 |
| Weight | 1.00 | 0.99 | 1.01 | 0.660 | 0.99 | 0.98 | 1.00 | 0.064 | 1.00 | 0.99 | 1.00 | 0.394 |
| Education (ref.: low) | | | |  |  |  |  |  |  |  |  |  |
| Middle | 0.87 | 0.57 | 1.32 | 0.506 | 0.88 | 0.73 | 1.07 | 0.195 | 0.77 | 0.63 | 0.95 | 0.015 |
| Higher | 0.78 | 0.50 | 1.23 | 0.282 | 0.78 | 0.61 | 1.02 | 0.067 | 0.72 | 0.57 | 0.91 | 0.005 |
| Smoking (ref.: never and ex-smoker) | | | | |  |  |  |  |  |  |  |  |
| Current | 2.12 | 1.51 | 2.98 | <0.001 | 1.45 | 1.22 | 1.73 | <0.001 | 2.08 | 1.65 | 2.62 | <0.001 |
| **Women** | **HR** | **95%CI** |  | **p-value** | **HR** | **95%CI** |  | **p-value** | **HR** | **95%CI** |  | **p-value** |
| **Model 1** |  |  |  |  |  |  |  |  |  |  |  |  |
| Grip | 0.90 | 0.86 | 0.94 | <0.001 | 0.92 | 0.90 | 0.94 | <0.001 | 0.96 | 0.94 | 0.98 | <0.001 |
| Height | 1.00 | 0.97 | 1.04 | 0.955 | 1.01 | 0.99 | 1.03 | 0.291 | 0.99 | 0.98 | 1.01 | 0.401 |
| Weight | 1.01 | 0.99 | 1.02 | 0.488 | 0.99 | 0.98 | 1.00 | 0.017 | 1.00 | 0.99 | 1.01 | 0.831 |
| **Model 2** |  |  |  |  |  |  |  |  |  |  |  |  |
| Grip | 0.90 | 0.86 | 0.94 | <0.001 | 0.92 | 0.90 | 0.94 | <0.001 | 0.97 | 0.95 | 0.98 | <0.001 |
| Height | 1.01 | 0.97 | 1.05 | 0.661 | 1.01 | 0.99 | 1.02 | 0.411 | 0.99 | 0.98 | 1.01 | 0.503 |
| Weight | 1.00 | 0.99 | 1.02 | 0.625 | 0.99 | 0.98 | 1.00 | 0.132 | 1.00 | 0.99 | 1.01 | 0.937 |
| Education (ref.: low) | | |  |  |  |  |  |  |  |  |  |  |
| Middle | 0.61 | 0.34 | 1.09 | 0.095 | 0.90 | 0.71 | 1.14 | 0.385 | 0.78 | 0.60 | 1.01 | 0.063 |
| Higher | 0.54 | 0.30 | 0.98 | 0.041 | 0.80 | 0.61 | 1.05 | 0.111 | 0.90 | 0.68 | 1.20 | 0.466 |
| Smoking (ref.: never and ex-smoker) | | | | |  |  |  |  |  |  |  |  |
| Current | 1.40 | 0.59 | 3.33 | 0.442 | 1.87 | 1.53 | 2.30 | <0.001 | 2.07 | 1.59 | 2.70 | <0.001 |

^a^ HR – hazard ratio, CI – confidence interval, SAHR – Study of Stress, Aging, and Health in Russia, MADT – the Study of Middle-Aged Danish Twins, LSADT – the Longitudinal Study of Aging Danish Twins, ELSA – the English Longitudinal Study of Ageing
