# Supplementary material for: Handgrip strength and its prognostic value for mortality in Moscow, Denmark, and England
Source: PLoS One. 2017 Sep 1;12(9):e0182684. doi: 10.1371/journal.pone.0182684 (PMC5580990; doi:10.1371/journal.pone.0182684)
Supplement: S2 Table — (DOCX) [file pone.0182684.s002.docx]

**S2 Table. Hazard ratios for grip strength per 1-kg increase in men and women in Moscow, Denmark, and England**

|  | **Men** |  |  |  | **Women** |  |  |  |
| --- | --- | --- | --- | --- | --- | --- | --- | --- |
|  | **HR^a^** | **95%CI** | | **p-value** | **HR** | **95%CI** | | **p-value** |
| **SAHR** |  |  |  |  |  |  |  |  |
| Model 1^b^ |  |  |  |  |  |  |  |  |
| Grip | 0.96 | 0.94 | 0.98 | <0.001 | 0.90 | 0.86 | 0.94 | <0.001 |
| Model 2 |  |  |  |  |  |  |  |  |
| Grip | 0.96 | 0.94 | 0.98 | <0.001 | 0.90 | 0.86 | 0.94 | <0.001 |
| Model 3 |  |  |  |  |  |  |  |  |
| Grip | 0.96 | 0.94 | 0.99 | 0.001 | 0.90 | 0.86 | 0.94 | <0.001 |
| Model 4 |  |  |  |  |  |  |  |  |
| Grip | 0.96 | 0.94 | 0.99 | 0.002 | 0.91 | 0.87 | 0.95 | <0.001 |
| Model 5 |  |  |  |  |  |  |  |  |
| Grip | 0.96 | 0.94 | 0.99 | 0.002 | 0.91 | 0.87 | 0.95 | <0.001 |
| Model 6 |  |  |  |  |  |  |  |  |
| Grip | 0.97 | 0.94 | 0.99 | 0.004 | 0.92 | 0.88 | 0.96 | <0.001 |
| **MADT and LSADT** | |  |  |  |  |  |  |  |
| Model 1 |  |  |  |  |  |  |  |  |
| Grip | 0.94 | 0.93 | 0.95 | <0.001 | 0.93 | 0.91 | 0.95 | <0.001 |
| Model 2 |  |  |  |  |  |  |  |  |
| Grip | 0.94 | 0.93 | 0.95 | <0.001 | 0.93 | 0.91 | 0.95 | <0.001 |
| Model 3 |  |  |  |  |  |  |  |  |
| Grip | 0.94 | 0.93 | 0.95 | <0.001 | 0.93 | 0.91 | 0.95 | <0.001 |
| Model 4 |  |  |  |  |  |  |  |  |
| Grip | 0.94 | 0.93 | 0.96 | <0.001 | 0.93 | 0.91 | 0.95 | <0.001 |
| Model 5 |  |  |  |  |  |  |  |  |
| Grip | 0.95 | 0.94 | 0.97 | <0.001 | 0.94 | 0.92 | 0.96 | <0.001 |
| Model 6 |  |  |  |  |  |  |  |  |
| Grip | 0.96 | 0.95 | 0.97 | <0.001 | 0.94 | 0.92 | 0.96 | <0.001 |
| **ELSA** |  |  |  |  |  |  |  |  |
| Model 1 |  |  |  |  |  |  |  |  |
| Grip | 0.97 | 0.96 | 0.99 | <0.001 | 0.96 | 0.94 | 0.98 | <0.001 |
| Model 2 |  |  |  |  |  |  |  |  |
| Grip | 0.98 | 0.97 | 0.99 | <0.001 | 0.96 | 0.95 | 0.98 | <0.001 |
| Model 3 |  |  |  |  |  |  |  |  |
| Grip | 0.98 | 0.97 | 0.99 | <0.001 | 0.97 | 0.95 | 0.98 | <0.001 |
| Model 4 |  |  |  |  |  |  |  |  |
| Grip | 0.98 | 0.97 | 0.99 | <0.001 | 0.97 | 0.95 | 0.99 | 0.001 |
| Model 5 |  |  |  |  |  |  |  |  |
| Grip | 0.99 | 0.97 | 1.00 | 0.014 | 0.97 | 0.96 | 0.99 | 0.010 |
| Model 6 |  |  |  |  |  |  |  |  |
| Grip | 0.98 | 0.97 | 0.99 | 0.004 | 0.98 | 0.96 | 1.00 | 0.026 |

^a^ HR – hazard ratio, CI – confidence interval, SAHR – Study of Stress, Aging, and Health in Russia, MADT – the Study of Middle-Aged Danish Twins, LSADT – the Longitudinal Study of Aging Danish Twins, ELSA – the English Longitudinal Study of Ageing

^b^ Model 1: Grip + height and weight;

Model 2: Grip + height, weight and education;

Model 3: height, weight, education, and smoking status

Model 4: Grip + height, weight, education, smoking status, and immediate recall

Model 5: Grip + height, weight, education, smoking status, immediate recall, and number of chronic conditions

Model 6: Grip + height, weight, education, smoking status, immediate recall, and self-rated health
